# Unilateral Nasopharyngeal Endoscopic Resection Type III: Technical Notes

**DOI:** 10.1002/lary.70378

**Published:** 2026-01-22

**Authors:** Carlo Conti, Gabriele Testa, Davide Mattavelli, Vittorio Rampinelli, Claudia Lodovica Modesti, Aurora Pinacoli, Alberto Schreiber, Cesare Piazza

**Affiliations:** ^1^ Department of Medical and Surgical Specialties, Radiological Sciences, and Public Health University of Brescia, School of Medicine Brescia Italy; ^2^ Unit of Otorhinolaryngology‐Head and Neck Surgery, ASST Spedali Civili of Brescia Brescia Italy

**Keywords:** head and neck surgery, mucosal melanoma, nasal endoscopy, nasopharyngeal endoscopic resection, oncologic surgery

## Abstract

The present video case report details technical notes of Nasopharyngeal Endoscopic Resection (NER) Type III, performed for a mucosal melanoma in a 71‐year‐old patient. Key surgical steps—such as achieving optimal exposure, employing Doppler‐guided internal carotid artery localization, and applying vascularized flap coverage—are outlined, demonstrating the precision and feasibility of this approach for treatment of these complex malignancies.

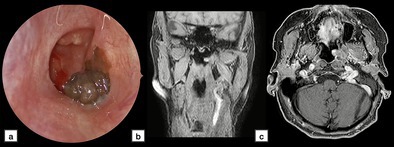

## 1 Introduction

Nasopharynx is a challenging surgical target due to its complex anatomy, deep location, and proximity to critical neurovascular structures. Nasopharyngeal endoscopic resection (NER) has revolutionized the treatment of nasopharyngeal lesions by offering increased magnification of the surgical field, improved esthetic outcomes, and reduced morbidity. In oncologic surgery, NER represents the mainstay of treatment for resectable recurrent nasopharyngeal carcinoma (rNPC), radioresistant non‐NPC, and benign lesions [1]. Castelnuovo et al. proposed a classification system with three Types of NER based on their extent [1]. Type III NER, the most commonly applied, entails resection of the nasopharyngeal mucosa together with the Eustachian tube (ET) and upper parapharyngeal space (UPPS).

This technical note describes the Type III NER in detail, focusing on procedural steps, anatomical landmarks, and perioperative management to improve surgical precision and safety.

## 2 Methods

**FIGURE 1:**
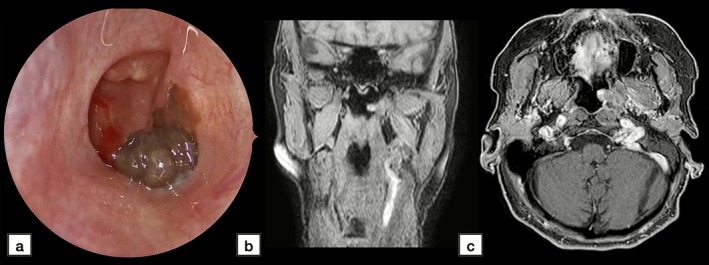
Endoscopic appearance (a) and magnetic resonance imaging (MRI) of the lesion (T1‐weighted sequence with contrast) in coronal (b) and axial (c) planes.

A 71‐year‐old woman presented with mucosal melanoma arising from the left pretubaric mucosa and posterior lateral nasal wall. Contrast‐enhanced magnetic resonance imaging (MRI) demonstrated a resectable lesion without significant PPS extension or critical relationships with the internal carotid artery (ICA) and skull base (Figure 1). A positron emission tomography with fluorodeoxyglucose revealed no regional or distant metastases. The case was discussed with the local multidisciplinary tumor board: considering the low tumor burden (cT3N0M0), the critical location (with the potential for ICA invasion or extension into the middle ear through the ET), and the limited evidence supporting the efficacy of neoadjuvant immunotherapy in sinonasal mucosal melanoma [2], a decision was made to proceed with radical surgery followed by adjuvant radiotherapy and immunotherapy. Since the disease was located on the lateral wall of the nasopharynx in close relationship with the ET, the approach required resection of its cartilaginous part (Type III NER).

Informed consent was obtained for the use of iconographic material and anonymized data.

## 3 Results

**FIGURE 2:**
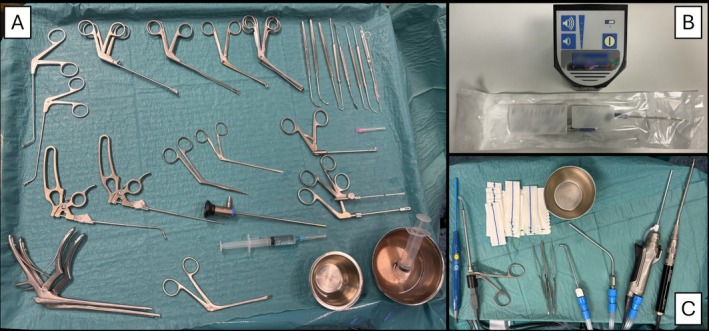
Surgical instrumentations, with cold instruments (A), doppler probe and unit (B), and powered and suction devices (C).

The surgical setting and instrumentations are represented in Figure 2.

### 3.1 Exposure of the Surgical Field

The first step involves preparing a surgical corridor for optimal visualization and working space. A partial middle turbinectomy, ethmoidectomy, and wide sphenoidotomy are essential to achieve adequate exposure of the pterygoid region. A medial maxillectomy Type B is performed: antrostomy is enlarged downward to the floor of the nasal cavity including the inferior turbinate, posteriorly until the palatine bone, and superiorly up to the floor of the orbit. The anterior border is represented by the lacrimal pathway. More extended medial maxillectomies may be required depending on the patient's anatomy and tumor lateral extension [3]. The posterior wall of the maxillary sinus and pterygoid area are then exposed, and the posterior maxillary sinus wall removed. The vertical portion of the palatine bone is resected exposing the greater and lesser palatine bundles that are coagulated at their entry point into the palatine foramen. The ipsilateral nasoseptal flap is then harvested with dissection of the pedicle into the pterygopalatine fossa (PPF) (i.e., resecting from medial to lateral the palatovaginal and Vidian arteries, and Vidian nerve), as recently described by Vinciguerra et al. [4]. Alternatively, a contralateral nasoseptal flap may be harvested to reduce technical difficulties and minimize interference in the surgical corridor, though this requires a longer route to the recipient site.

Subsequently, the content of the PPF, including the flap pedicle, is lateralized, bringing the pterygoid process into view. When an ipsilateral nasoseptal flap is not harvested or the tumor extends laterally into the infratemporal fossa, it is crucial to identify, ligate, and cut the maxillary artery (MA) or its terminal branches. The MA is identified through blunt dissection into the infratemporal fatty tissue, carefully isolated from the surrounding structures, and secured with metallic clips distally. Then, the second surgeon applies traction and straightens the vessel using a ball probe, while the first surgeon progressively cuts the artery. The PPF can be entirely removed taking care not to damage the second branch of the trigeminal nerve at the level of the foramen rotundum.

**FIGURE 3:**
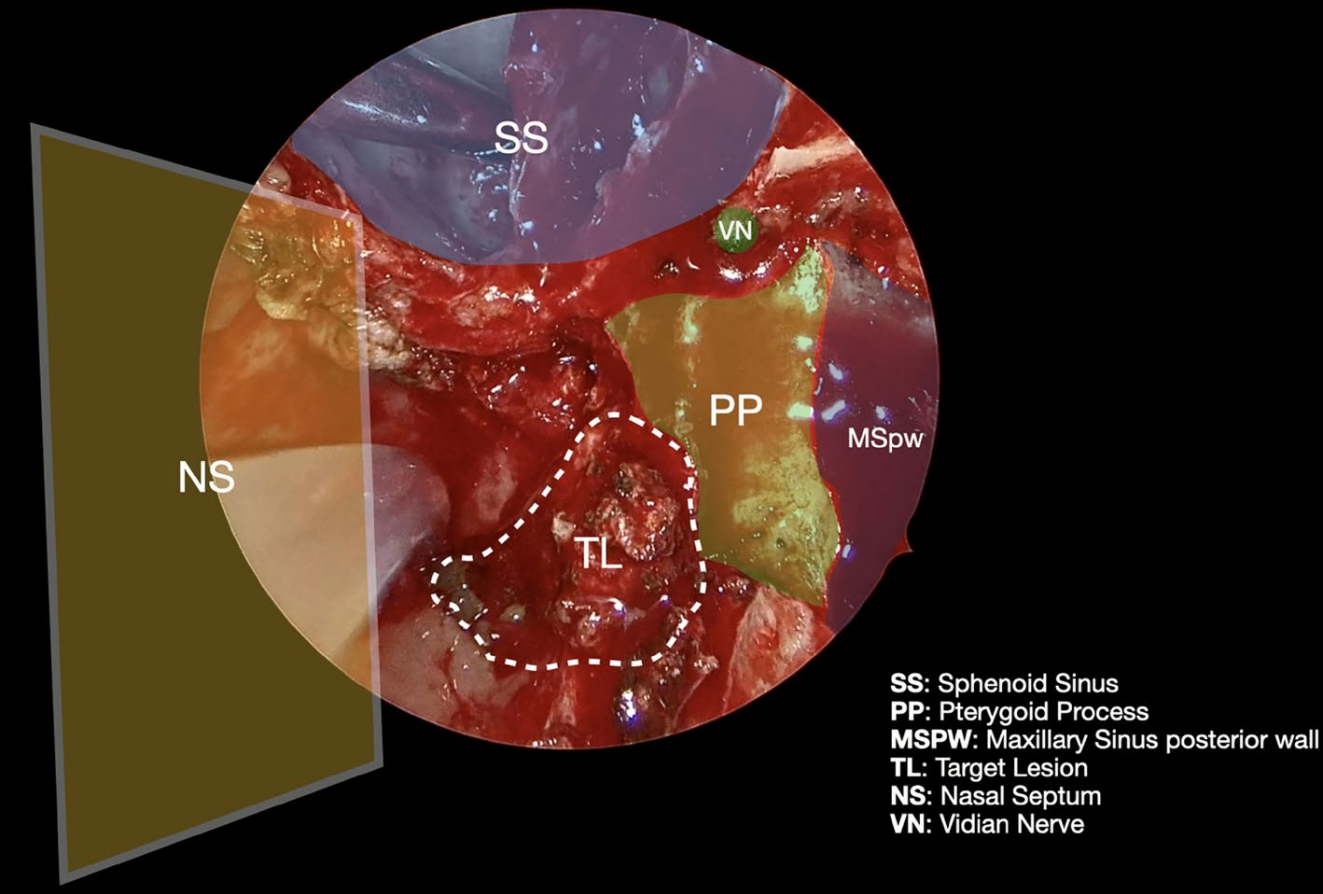
View of the target lesion (TL) after the exposure phase consisting of the medial maxillectomy and removal of the posterior third of the nasal septum (NS), with exposure of the Vidian nerve foramen (VN), sphenoid sinus (SS), pterygopalatine process (PP), and the posterior wall of the maxillary sinus (MSPW).

The posterior fourth of the nasal septum is removed by making an incision at the rostrum‐vomer joint. The rostrum is subsequently excised, and a complete trans‐rostral sphenoidotomy is performed. Two flexible plastic tubes are then entered through the nose and passed down to the oropharynx and oral cavity to retract anteriorly the soft palate. This maneuver helps to visualize the posterior wall of the nasopharynx, and better delineate the inferior margin of resection. The exposure phase is completed when the pterygoid process, the Vidian foramen and canal, the entire vault of the nasopharynx, and both sphenoid sinuses are clearly visible (Figure 3).

### 3.2 Nasopharyngeal Resection

**FIGURE 4:**
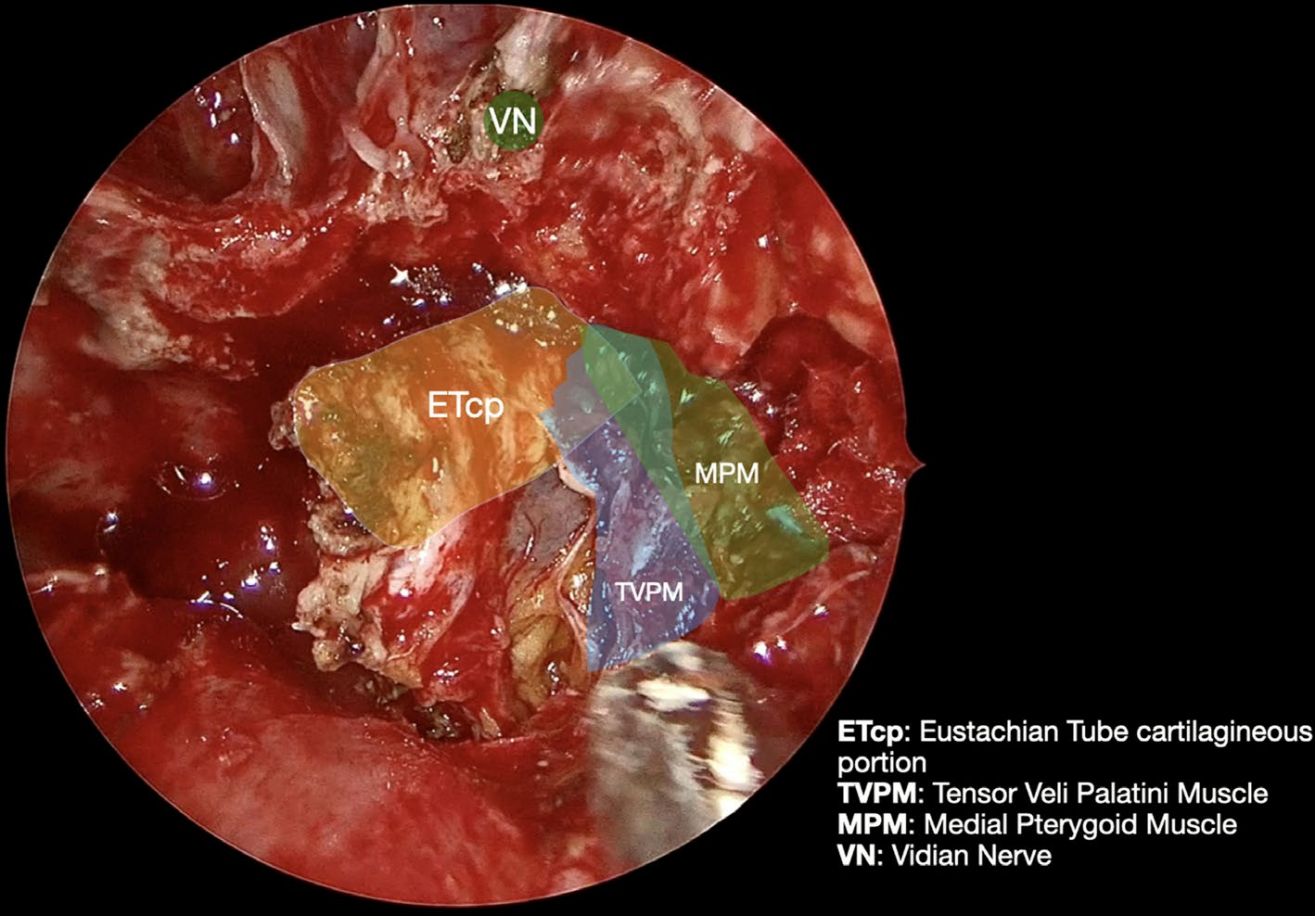
View after resection of the pterygoid process below the Vidian nerve foramen (VN); the cartilaginous portion of the Eustachian tube (ETcp) is exposed. Anteriorly, the muscular bundles of the medial pterygoid (MPM) and tensor veli palatini muscles (TVPM) can be seen.

**FIGURE 5:**
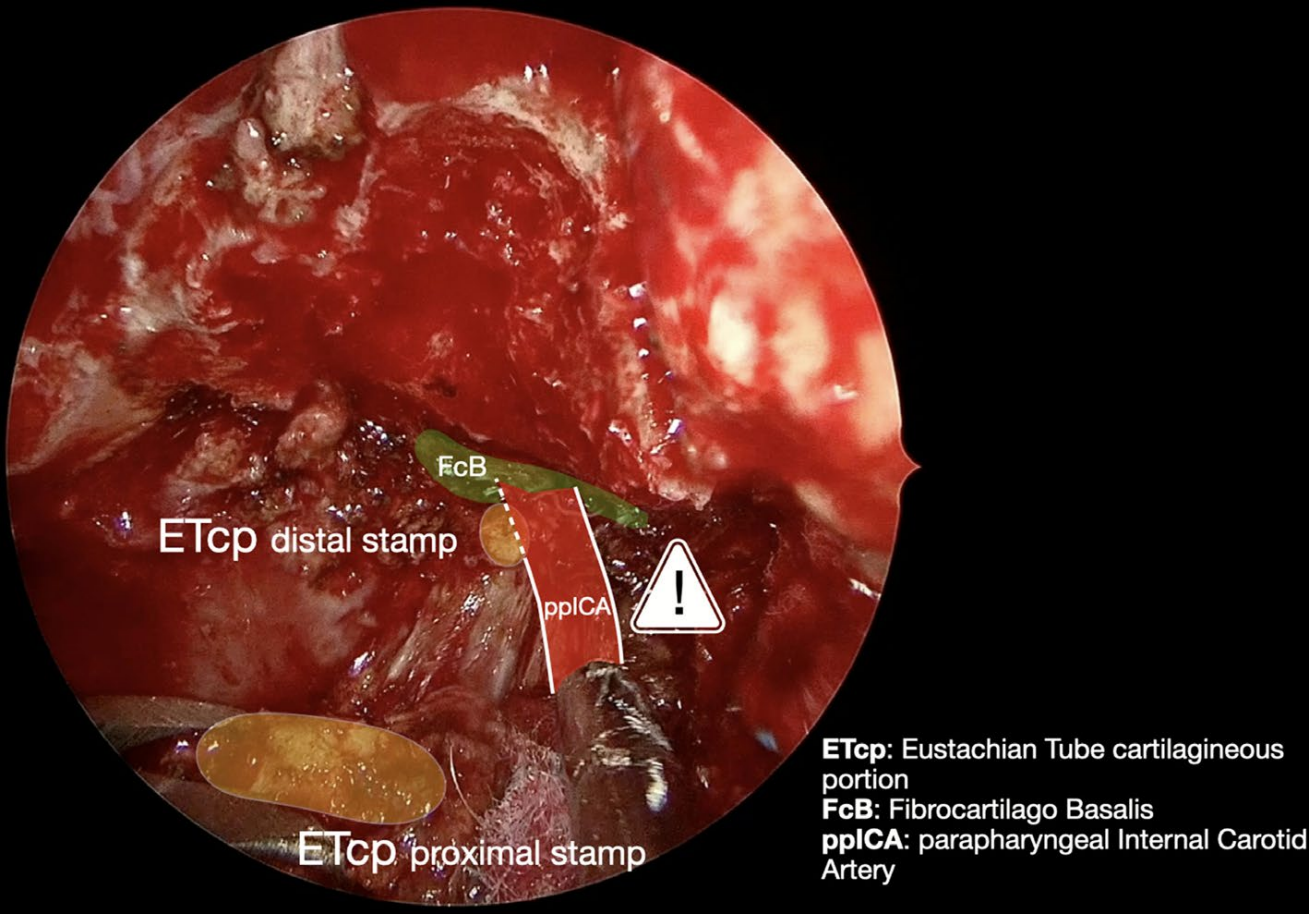
View after resection of the cartilaginous portion of the Eustachian Tube (ETcp). Just posterior to the distal stamp of the tube (yellow, small circle), the parapharyngeal tract of the internal carotid artery (ppICA, red) can be encountered in its last extracranial tract while entering the fibrocartilago basalis (FcB, green) of the foramen lacerum.

A mucosal incision is made to outline the lesion at the nasopharyngeal vault and posterior wall, maintaining a safe margin from the tumor. If needed, the inferior incision may be performed via a transoral route under endoscopic guidance. On the lateral wall the inferior incision is extended anteriorly including a portion of the soft palate mucosa running below the ET and ending at the pretubaric level. The floor of the sphenoid is drilled until the junction between the middle and lower clivus is reached. While the second surgeon applies traction on the specimen, the nasopharyngeal mucosa and longus capitis muscle are resected from the vertebral bony layer, in a cranio‐caudal and latero‐medial direction. In the present case, the posterior and midline dissection were limited to a mucosectomy, as the tumor was confined to the pretubaric area, making it unnecessary to reach the vertebral plane, which can result in significant postoperative pain. The mucosa is then detached from the medial pterygoid plate to fully expose the anterior and medial aspect of the pterygoid. In this way the incision is then reconnected with the inferior margin already outlined on the soft palate. The medial pterygoid plate is then drilled vertically from the floor of the nasal cavity up to the Vidian foramen and canal, which mark the superolateral boundary of the resection. The Vidian nerve serves as a crucial landmark, as it leads to the medial genu of the ICA and to the foramen lacerum. The pterygoid fossa (PF) is entered and the insertion of the medial pterygoid muscle (MPM) exposed. Posteromedial to the MPM, the vertical fibers of the tensor veli palatini muscle (TVPM) can be identified (Figure 4). The lateral pterygoid plate represents an important landmark for the carotid foramen that lies on the same parasagittal plane. Therefore, it is advisable to keep it untouched unless the dissection needs to be extended far lateral into the UPPS. The MPM and TVPM are sectioned inferiorly at the level of the nasal floor and soft palate, until the fatty tissue of the UPPS, located lateral to the velar muscles, is exposed. At this point, the oblique fibers of the levator veli palatini muscle (LVPM) are identified and sectioned at the palatal level. It is advisable to use a Doppler probe during this step to assess the position of the parapharyngeal segment of the ICA (ppICA), as traction may cause the artery to shift medially and anteriorly from its radiological location. By continuing to drill the pterygoid root medially and inferiorly to the Vidian canal, the cartilaginous portion of the ET is identified. The most critical step of the procedure is represented by the posterolateral margin of resection since the ppICA lies in this area just posterior to the ET. The best way to identify the ppICA is to follow the tensor–vascular–styloid fascia in the fatty tissue lateral to the TVPM [5] even if this plane is easier to identify in naïve patients since the presence of scar tissue in an irradiated field may hamper its identification. In this step, the use of a Doppler probe is mandatory to monitor the location of the ICA while dissecting and cutting tissues (Figure 5). The cartilaginous portion of the ET (cpET) must be resected at its isthmus (i.e., the junction between the cartilaginous and bony tracts of the ET) using a monopolar scalpel with the tip angled at 45° in a latero‐medial direction or using cold instrumentation, thus minimizing the risk of accidental damage to the ppICA. Once the cpET is sectioned, the deep posterior margin at the clival and vertebral level is connected, completing the posterolateral margin, while continuously monitoring the position of the ppICA with the Doppler probe. The surgical specimen is extracted through the nose and properly oriented. It must include, from anterior to posterior, the mucosa of the lateral wall of the nasopharynx, the MPM at its insertion into the PF, the TVPM and LVPM, the cpET, prevertebral soft tissues, and the mucosa of the posterior wall and vault of the nasopharynx.

### 3.3 Coverage of the Surgical Field With a Vascularized Flap

The nasoseptal flap is placed over the resection site, particularly the exposed spheno‐clival bone. In previously irradiated patients or more extensive resections, a temporoparietal fascial flap (TPFF) is usually preferred. Ensuring close adhesion of the flap to the surgical bed is essential to prevent dead space, with resorbable materials aiding in its positioning. Nasal packing is usually left in place for 2–3 days. Due to ET resection, a myringotomy with trans‐tympanic drainage tube placement is mandatory to prevent chronic effusive otitis (Video 1).

**VIDEO 1:** Unilateral nasopharyngeal endoscopic resection (NER) Type III for mucosal melanoma of the left nasopharynx—video technical notes and case report. Video content can be viewed at https://onlinelibrary.wiley.com/doi/10.1002/lary.70378.

## 4 Discussion

Even though the indication for this procedure in cases of mucosal melanoma remains debated, given the tumor's tendency to metastasize and to develop satellite lesions distant from the primary site, we elected to proceed with surgery in this particular patient because of the very localized nature of the disease, the favorable anatomy, and the already compromised function of the right Eustachian tube.

We chose to present this case instead of a more typical recurrence of nasopharyngeal carcinoma because, in this non‐irradiated setting, the anatomy is much clearer in the absence of fibrosis and synechiae.

The most common indication for NER type III remains salvage surgery for recurrent nasopharyngeal carcinoma. However, it may also be considered as a primary treatment option for selected non‐NPC malignant tumors arising from the nasopharynx or upper parapharyngeal space—such as minor salivary gland tumors or for benign lesions originating in this region, including schwannomas and angiofibromas. This approach is contraindicated in cases of carotid space invasion, extensive skull base involvement (e.g., clivus or petrous bone), intracranial extension, or in lesions exhibiting an infiltrative growth pattern beyond the pharyngobasilar fascia [1]. NER Type III presents a unique set of challenges, particularly in the management of the ICA. It is paramount to perform this procedure in a hospital equipped with interventional neuroradiology and having a close collaboration with anesthesiologists due to the potential, albeit rare, event of a carotid blowout [6].

There are several aspects the surgeon must keep in mind while approaching the ICA, the most relevant being:

− Preoperatively, its course must be thoroughly studied on imaging; in case a high risk of ICA injury is predictable, a balloon occlusion test is advisable;
− The Doppler probe is a fundamental tool to confirm the ICA location during dissection, while surgical navigation may be imprecise, as this vessel can shift during the procedure due to traction on the specimen, and its accuracy tends to decrease the deeper the surgical field.

The flap coverage is essential in case of exposure of the ICA wall and over the clival and sphenoidal bone to minimize the risk of post‐treatment osteomyelitis and/or osteoradionecrosis, which could potentially lead to a delayed carotid blowout. The choice of the flap depends on patient's features. If available, the nasoseptal flap is the most convenient option. In contrast, when this flap is no longer available or the patient has a high risk for osteomyelitis and/or osteoradionecrosis, the TPFF becomes an excellent option. It can be translocated into the nasal cavity through the infratemporal fossa at the level of the posterior wall of the maxillary sinus using a percutaneous tracheostomy introducer set. This flap is highly vascularized and bulky, and it is particularly efficient in guiding the healing process in irradiated surgical fields [7].

Finally, the positioning of a trans‐tympanic drainage tube is mandatory to avoid hearing loss and the stagnation of secretions in the middle ear that could be the initial trigger for a possible osteomyelitis.

In this case report, surgical resection was performed upfront in view of the poor radiosensitivity of mucosal melanoma. Final histopathological examination confirmed pT3 mucosal melanoma with free resection margin. Postoperative course was uneventful. Adjuvant radiation (66 Gray in 33 fractions) followed by immunotherapy was delivered. The patient is currently alive without disease 9 months after the end of treatment.

## Funding

The authors have nothing to report.

## Conflicts of Interest

The authors declare no conflicts of interest.
